# *In utero* methadone exposure permanently alters anatomical and functional connectivity: A preclinical evaluation

**DOI:** 10.3389/fped.2023.1139378

**Published:** 2023-02-23

**Authors:** Eric M. Chin, Yuma Kitase, Nethra K. Madurai, Shenandoah Robinson, Lauren L. Jantzie

**Affiliations:** ^1^ Department of Neurodevelopmental Medicine, Phelps Center for Cerebral Palsy and Neurodevelopmental Medicine, Kennedy Krieger Institute, Baltimore, MD, United States; ^2^ Department of Neurology, Johns Hopkins University School of Medicine, Baltimore, MD, United States; ^3^ Department of Pediatrics, Johns Hopkins University School of Medicine, Baltimore, MD, United States; ^4^ Department of Pediatrics, Division of Neonatal-Perinatal Medicine, Johns Hopkins University School of Medicine, Baltimore, MD, United States; ^5^ Department of Neurosurgery, Division of Pediatric Neurosurgery, Johns Hopkins University School of Medicine, Baltimore, MD, United States

**Keywords:** prenatal opioid exposure, methadone, functional connectivity, white matter microstructure, neurodevelopment

## Abstract

The opioid epidemic is an ongoing public health crisis, and children born following prenatal opioid exposure (POE) have increased risk of long-term cognitive and behavioral sequelae. Clinical studies have identified reduced gray matter volume and abnormal white matter microstructure in children with POE but impacts on whole-brain functional brain connectivity (FC) have not been reported. To define effects of POE on whole brain FC and white matter injury in adult animals, we performed quantitative whole-brain structural and functional MRI. We used an established rat model of POE in which we have previously reported impaired executive function in adult rats analogous to persistent neurocognitive symptoms described in humans with POE. Pregnant Sprague-Dawley rat dams received continuous methadone (12 mg/kg/day) vs. saline infusion for 28 days via osmotic mini-pumps, exposing rats to pre- and postnatal opioid until weaning. At young adult age (P60), POE and saline exposed offspring underwent *in vivo* MRI included diffusion tensor imaging and functional MRI (fMRI). Results indicate that fractional anisotropy (FA) was decreased in adult animals with POE [*n* = 11] compared to animals that received saline [*n* = 9] in major white matter tracts, including the corpus callosum (*p* < 0.001) and external capsule (*p* < 0.01). This change in FA was concomitant with reduced axial diffusivity in the external capsule (*p* < 0.01) and increased radial diffusivity in the corpus callosum (*p* < 0.01). fMRI analyses reveal brainwide FC was diffusely lower in POE (*p* < 10^−6^; 10% of variance explained by group). Decreased connectivity in cortical-cortical and cortico-basal ganglia circuitry was particularly prominent with large effect sizes (Glass's Δ > 1). Taken together, these data confirm POE reduces brainwide functional connectivity as well as microstructural integrity of major white matter tracts. Altered neural circuitry, dysregulated network refinement, and diffuse network dysfunction have been implicated in executive function deficits that are common in children with POE. FC may serve as a translatable biomarker in children with POE.

## Introduction

The opioid epidemic is a public health crisis (1, 2). The National Institutes of Health (NIH) has deemed opioid misuse a national health emergency (3, 4), and efforts to address the opioid crisis are major priorities of the US congress (5, 6), March of Dimes Foundation (5, 7), and World Health Organization (8). Centers for Disease Control and Prevention (CDC) estimate the total economic burden of opioid misuse to be 78.5 billion USD annually, underscoring the enormous impact on health, social and financial well-being (3, 9, 10). Pregnant women and children are often overlooked in public health efforts to address the opioid crisis. Indeed, the incidence of substance misuse during pregnancy and its negative impact on postnatal outcomes is a critical threat to pediatric and adult health (11). Thus, there is an immediate need to define the full spectrum of adverse outcomes associated with prenatal opioid exposure (POE) (1).

The incidence of substance misuse during pregnancy and its negative impacts on postnatal outcomes requires intense research efforts (11). The increased prevalence of opioid use disorder (OUD) in pregnant people is paralleled by a staggering increase in neonatal opioid withdrawal syndrome (NOWS) (12–15). NOWS is a well-recognized clinical syndrome associated with POE. It has risen 5-fold in the past decade. Specifically, in the USA NOWS occurs in ∼5.8 infants in every 1000-hospital births, accounting for an estimated 1.5 billion dollars in hospital charges, the majority of which is incurred by Medicaid, in addition to the cumulative individual, familial and societal burdens (1, 13, 16–18). Maryland has one of the highest rates of OUD recorded at infant delivery and these numbers have more than quadrupled from 1999 to 2014 similar to national statistics (19).

While NOWS is a well-defined clinical syndrome, the potential for long-term damage to the developing brain due to opioid medications remains a serious and poorly understood concern. Recently, there is greater appreciation that the adverse effects of POE on neurodevelopment extend far beyond the symptoms of NOWS. Not all infants with POE who are at risk for brain injury exhibit withdrawal symptoms (20–22). In line with clinical practice guidelines, OUD is typically treated with methadone or buprenorphine during pregnancy as a safer alternative to abstinence or withdrawal. However, the safety of opioid maintenance treatment during pregnancy, including the use of methadone and buprenorphine to manage OUD, has been defined by studies with limited evaluation of postnatal sequelae, with no randomized control trials that included imaging or long-term follow-up on the exposed children (5, 23–28).

Here, we build on a growing body of literature examining chronic changes to brain structure and function caused by POE (20, 29–37). We hypothesized that methadone would be toxic to developing neural cells resulting in structural and functional brain injury. We expected that *in utero* methadone exposure would cause disruption of white matter microstructure and deficits in functional connectivity—manifestations of sustained neural network dysfunction. Using state-of-the-art preclinical magnetic resonance imaging (MRI), including diffusion tensor imaging (DTI) and functional connectivity using functional MRI (FC/fMRI), we examined neural networks and major white matter tracts essential to cognition.

## Methods

### Animals

Sprague-Dawley rat dams and litters were maintained in a temperature and humidity-controlled facility with food and water available *ad libitum*. A 12-hour dark/light cycle was maintained for all animals with lights on at 0800 h. All experiments were performed in strict accordance with protocols approved by the institutional Animal Care and Use Committee (ACUC) at the Johns Hopkins University. Protocols were developed and performed consistent with National Research Council and ARRIVE guidelines (38). Litter size was similar between methadone-exposed and saline-exposed litters, with no differences in maternal weights. As previously published (39–41), pup weights were significantly lower in methadone-exposed litters as compared to saline-exposed litters. For each experiment described, the data represents true n (individual rats). Each rat fetus has its own placenta and thus, represents an individual maternal-placental-fetal unit. Accordingly, 1 fetus/pup is considered a singular experimental unit consistent with published norms. However, for every experiment and outcome measure, we used offspring from at least 4 different dams and litters per condition to control for the potential of litter effects. There was no difference in maternal care, including on nest and off nest activities observed between groups. Male and female offspring were used in every outcome measure and in approximately equal numbers where possible.

### Methadone exposure

**Figure 1:**
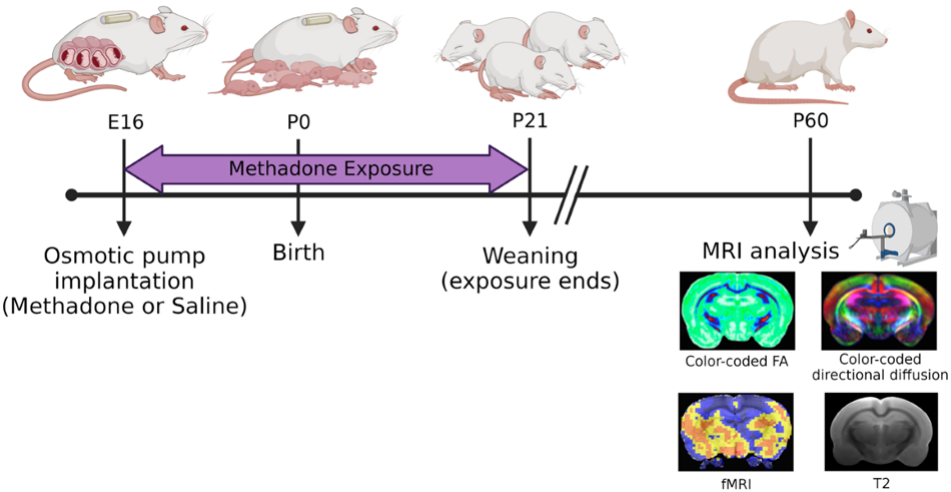
Experimental paradigm. This timeline highlights key study timepoints (pump implantation, birth, weaning, and *in vivo* brain imaging in young adulthood).

Per previously published methods, on embryonic day 16 (E16), osmotic mini pumps (ALZET, Cupertino, California) were implanted subcutaneously in the nape of the neck of pregnant dams for 28 days of continuous methadone (12 mg/kg/day infused at 0.25 µl/h flow rate) or sterile saline infusion (Figure 1) (39–41). Methadone is a synthetic, long-acting, µ-opioid receptor agonist that readily crosses the placenta and blood-brain barrier. Specifically, following induction and maintenance of anesthesia with inhaled isoflurane, dams underwent minipump placement with a 1.5 cm transverse skin incision followed by careful blunt dissection of the subcutaneous space. Osmotic pumps were prefilled and primed prior to insertion. Dams were carefully monitored after closure with 2–3 sutures following the procedure for full recovery. Rat pups were born at E22/postnatal day 0 (P0) following completion of gestation and remained with their dams. Pups continued to receive methadone or saline through the maternal milk supply until weaning on P21 (39–41).

### Imaging

**Figure 2:**
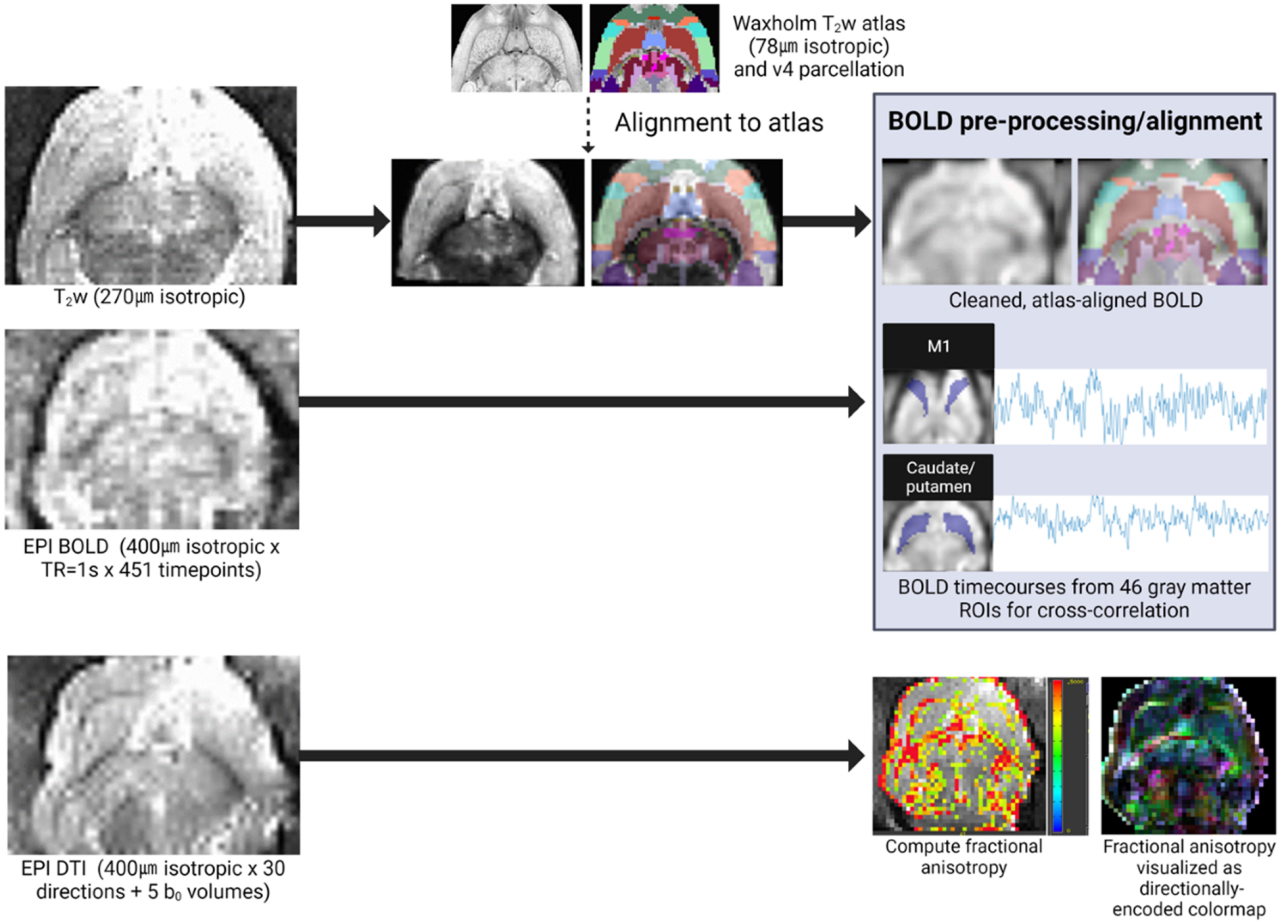
Quantitative MRI analysis pipeline. *In vivo* imaging included a T_2_-weighted anatomical sequence (for alignment to the Waxholm reference atlas), BOLD-weighted fMRI, and DTI. Individual fMRI scans were pre-processed and aligned to the reference atlas for extraction of ROI-specific BOLD timecourses for cross-correlation in functional connectivity analyses. Individual DTI scans were manually segmented (extracting corpus callosum and external capsule ROIs), and these white matter ROI masks were used to compute diffusion scalars (e.g., FA pictured here).

*In vivo* imaging was performed on P60, (young adult age equivalent) using an 11.7 T scanner (Bruker BioSpec, Billerica, MA; Figure 2). Rats were sedated with dexmedetomidine for multisequence acquisition using a volumetric head coil. Our imaging protocol included a high-resolution fat-suppressed T_2_-weighted anatomical sequence (0.27 mm isotropic resolution; 2 averages), BOLD-weighted fMRI [0.4 mm isotropic with TR = 1000 ms × 451 volumes, TE minimized (4.5 ms)], and high-resolution diffusion imaging (0.4 mm isotropic × 30 directions at b = 1,000 and 5 b_0_ volumes)—all with whole-brain coverage.

### Microstructure analysis (DTI)

We performed a quantitative DTI analysis of white matter microstructure alterations in POE. We selected white matter regions of interest (ROI) *a priori* that have been implicated in functional outcome and cognition (corpus callosum and external capsule). As we have performed previously (39, 42–47), ROIs were traced by an observer masked to experimental conditions and analyzed using Bruker's Paravision 6.1 imaging software (Billerica, MA). In brief, fractional anisotropy (FA), axial diffusivity (*λ*_1_), and radial diffusivity $\left(\frac{{\;\lambda }_{2}+{\;\lambda }_{3}}{2}\right)$ scalar maps were computed, and means were calculated individually for each ROI. For bilateral neuroanatomical ROIs, scalar means were acquired on each side and averaged per ROI. Two scans (both in the Saline group) were excluded from analysis—one due to poor field of view coverage and one due to severe motion-related artifact.

### Functional MRI (fMRI) analysis

Resting state functional imaging data were pre-processed using AFNI version 20.1.06 (Bethesda, MD). T_2_-weighted anatomical images were pre-processed (skullstripped using the AFNI @NoisySkullStrip function) and intensity-normalized (3dUnifize function). Non-linear warp transformations to the Waxholm Atlas T_2_-weighted reference image were computed for T_2_-weighted anatomical and BOLD-weighted fMRI images simultaneously (@AnimalWarper function, feature_size = 0.05 mm) (48). This transformation, as well as pre-processing, were applied to BOLD-weighted images using the afni_proc.py function. We employed stringent *a priori* artifact correction to mitigate anticipated artifacts including artifactual spatial distortion (mitigated using non-linear alignment as above), cardiorespiratory artifact, and effects of head motion. In particular, additional pre-processing steps used within afni_proc.py removed pre-steady state volumes (first 2 TRs), applied slice timing correction, applied despiking, aligned BOLD volumes to each other, applied a Gaussian blur (0.8 mm full width at half maximum), applied outlier censoring (rejecting BOLD volumes during which more than 5% of brain voxels were outliers), low-pass filtering (0.08 Hz cutoff) to mitigate cardiac/respiratory artifact, and regression of nuisance variables (6 axes of head motion as well as their first time derivatives). We also utilized customized quality control procedures to only include scans with adequate BOLD-atlas alignment and with gray matter temporal signal-to-noise consistently above 100 (more typically exceeding 200). One scan (Saline group) was excluded from analysis due to poor field of view coverage.

Gray matter regions of interest (ROIs) were selected *a priori* from the version 4 Waxholm Atlas (accessed via https://www.nitrc.org/projects/whs-sd-atlas). Cortical, and subcortical gray matter ROIs were selected that (1) were expected to lie within the imaging field of view; (2) were related to sensory, motor, pain, affective, or cognitive functioning; (3) and were at least 15 voxels in size when resampled into the 0.4 mm isotropic imaging matrix used in this study. In total, 46 ROIs were examined in terms of region-to-region functional connectivity (see Supplemental Data Sheet for details). Functional connectivity was computed on an individual scan level as the Fisher Z-transformed Pearson correlation coefficient of ROIs' voxelwise mean BOLD signal timecourses.

### Statistical analysis

Diffusion data are represented as mean ± the standard error of the mean (SEM). Data was tested for normality using the Shapiro-Wilk test. When data for both groups was normal (Shapiro-Wilk *p* > 0.05), statistical differences were established with two-tailed Student's *t*-tests. When either demonstrably deviated from normality (Shapiro-Wilk *p* < 0.05; Saline RD), we conservatively employed the non-parametric Mann-Whitney test. In either case, *p* < 0.05 in a two-tailed test was considered statistically significant.

In fMRI analyses, we directly tested our hypothesis of diffuse, global FC changes across the brain by examining patterns of group x edge differences using a Type III ANOVA—attempting to distinguish (1) connectivity patterns that are common across all scans (main effect of edge), (2) brain-wide differences in connectivity magnitude between study groups (main effect of group), and (3) differences between groups in specific network connections (interaction of edge x group).

ROI-to-ROI connections were also examined individually using a non-parametric rank sum test with multiple comparisons correction performed using the Benjamini-Hochberg procedure (false discovery rate = 0.05) due to the large number of imaging features compared (FC for each of 46*45/2 = 1,035 ROI-to-ROI connections). In addition to binarized hypothesis testing, we additionally examined group differences in terms of standardized effect size (Glass's Δ assessing differences between group means in units of the standard deviation of FC_Saline_) to descriptively define patterns of altered FC.

GraphPad Prism 9.3.1 software and MATLAB version 2022a (MathWorks, Natick, MA) were used to perform statistical analysis.

## Results

### Microstructural analyses

**Figure 3:**
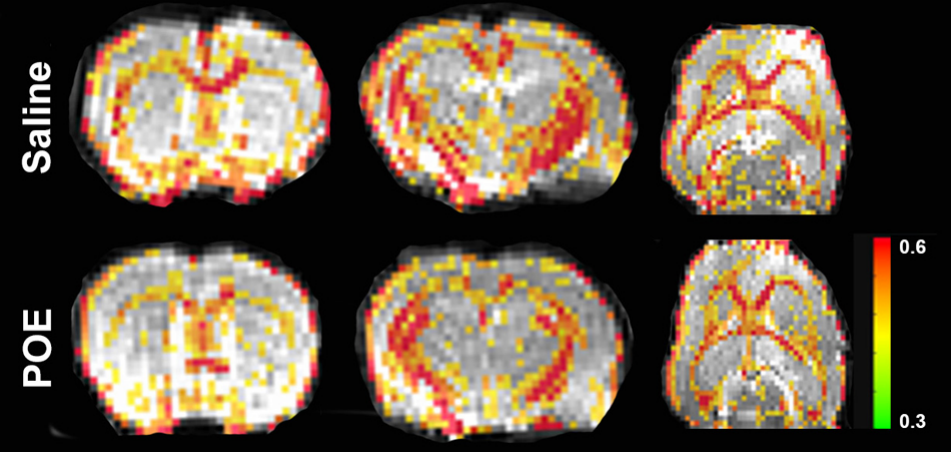
White matter microstructure alterations in POE: fractional anisotropy maps. These are sections of whole-brain fractional anisotropy maps in one representative Saline group scan (***top row***) and in one representative POE scan (***bottom row***). Two coronal sections (***first two columns***) and an axial section (***last column***) are included here. In each panel, the grayscale background is a raw b = 0 image; the superimposed colored voxels indicate fractional anisotropy (FA) for white matter voxels (voxels with FA > 0.3). The color of the white matter voxel indicates the FA value (from high [0.6+; red] to low [0.3; green]). Note that high-FA regions of large white matter tracts overall appear to be wider (spanning a greater diameter within each tract) and extend further along the length of each tract.

**Figure 4:**
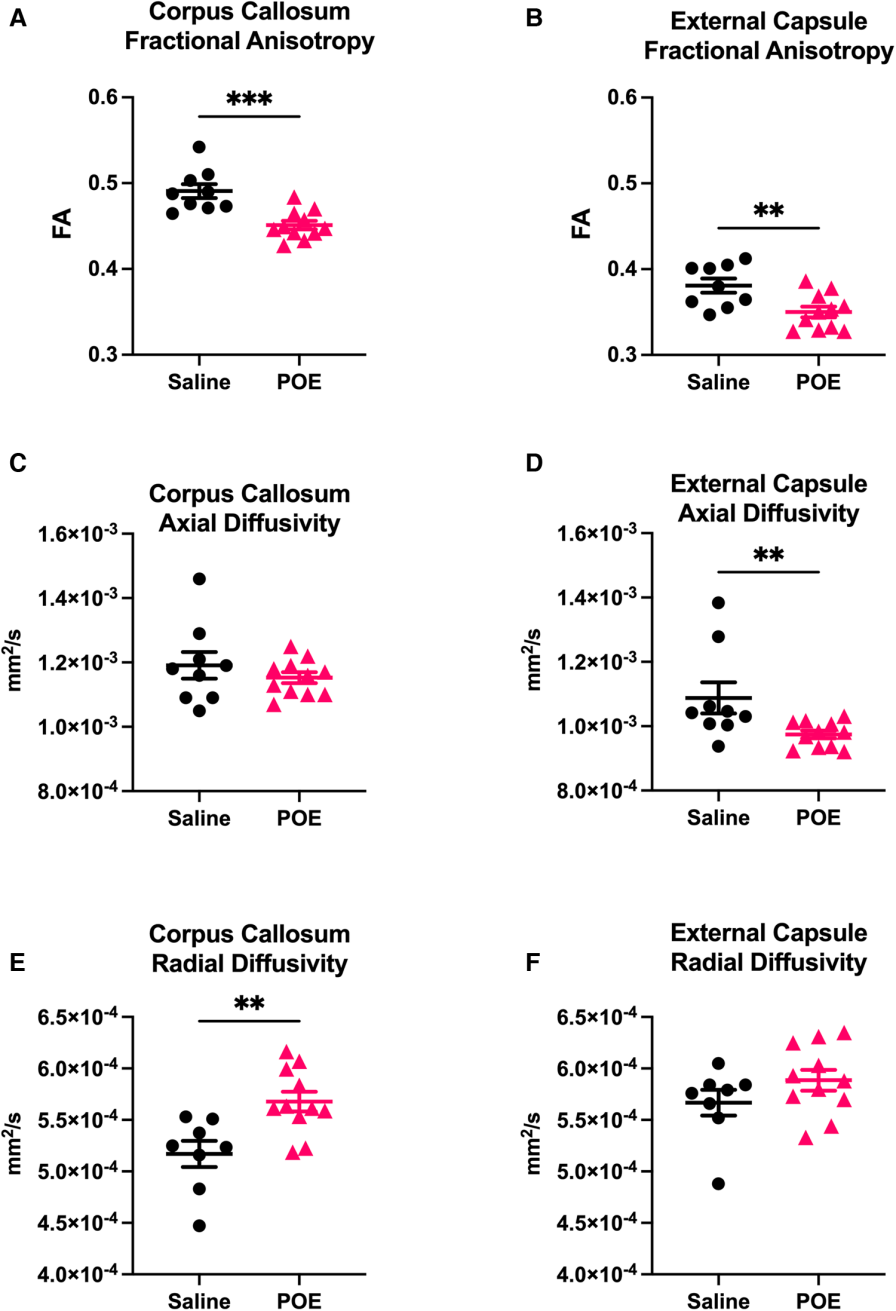
White matter microstructure alterations in POE: groupwise comparison of diffusion tensor metrics. Diffusion tensor metrics assess permeability to water flow (diffusivity) along (axial) vs. perpendicular to (radial) white matter tracts. Metrics were examined in two regions of interest (ROIs): corpus callosum (***left column:* A,C,E**) and external capsule (***right column:* B,D,F**). In each plot, individual values (black circles [Saline] vs. gray triangles [POE]) are plotted as well as group statistics (mean ± SEM). Fractional anisotropy (FA; **A,B**) can be considered a measure of microstructural flow selectivity (near one when axial diffusivity [AD] >> radial diffusivity [RD]; near zero when AD ≈ RD). Note that FA is decreased in POE in both ROIs. This appears to be attributable to decreased AD and increased RD in both ROIs, though differences are most statistically significant for AD in external capsule and for RD in corpus callosum.

Fractional anisotropy (FA) was decreased in POE compared to saline controls (Figure 3), including in both the corpus callosum (Saline: 0.491 ± 0.008 vs. POE: 0.451 ± 0.005; *p* < 0.001) and in the external capsule (Saline: 0.381 ± 0.008 vs. POE: 0.350 ± 0.006; *p* < 0.01; Figures 4A,B). High-FA regions of large white matter tracts overall appeared to be wider (spanning a greater diameter within each tract) and to extend further along the length of each tract (Figure 3).

As FA is a measure of microstructural flow selectivity (near one when axial diffusivity [AD] >> radial diffusivity [RD]; near zero when AD ≈ RD), decreased AD or increased RD can both contribute to differences in FA and be associated with axonal injury and impaired myelination. AD was significantly decreased in the external capsule (Saline: 1.1 × 10^−3 ^± 4.8 × 10^−5^ vs. POE: 0.9 × 10^−3 ^± 1.2 × 10^−5^; *p* < 0.01) and trended lower in the corpus callosum (Figures 4C,D). RD was increased in the corpus callosum (Saline: 5.2 × 10^−4 ^± 1.2 × 10^−5^ vs. POE: 5.7 × 10^−4^ ± 9.5 × 10^−6^; *p* < 0.01) and trended higher in the external capsule (Saline: 5.7 × 10^−4 ^± 1.2 × 10^−5^ vs. POE: 5.9 × 10^−4 ^± 1.9 × 10^−5^; *p* = 0.2; Figures 4E,F).

### Functional connectivity

**Figure 5:**
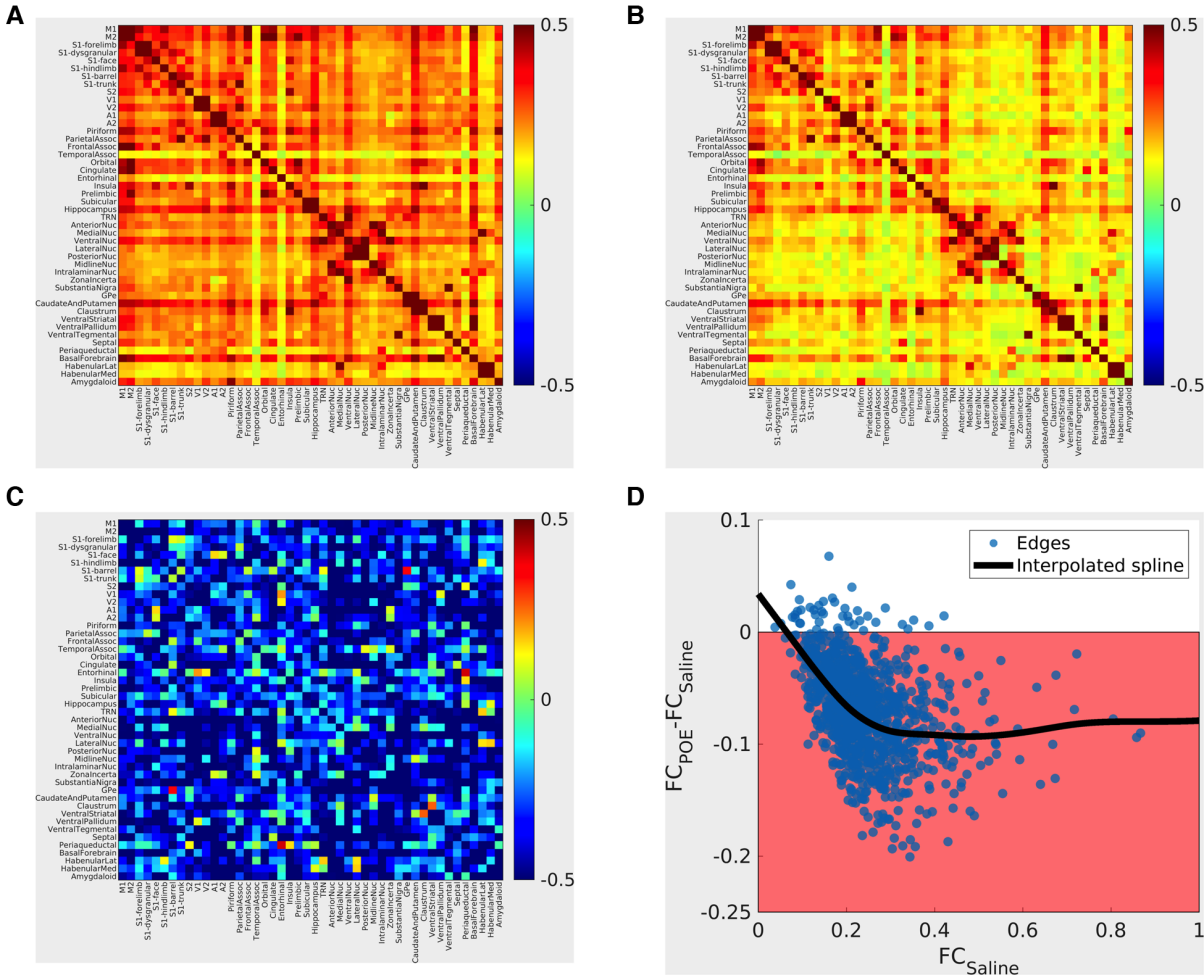
Functional connectivity alterations in POE. *Top panels: Group mean cross-correlation (functional connectivity) matrices*. Heatmaps (**A**: saline group; **B**: methadone group) summarize functional connectivity profiles seen in each group—each row and each column correspond to a gray matter ROI, and the color of the voxel at the intersection of the row/column indicates the functional connectivity seen between the two ROIs (warm colors = positive correlation; cool colors = negative correlation). “Strong” (high FC) connections seen nearly all consisted of positive correlations and that “strong” connections were seen in both groups, for example, within sensorimotor cortical networks and between thalamic nuclei. ***Bottom left panel* C*: Between-group differences*** are pictured in terms of standardized effect size (Glass's Δ; differences in group means in units of Saline standard deviation). Highly positive values (warm colors) indicate FC_POE _> FC_Saline_, and highly negative values (cool colors) indicate FC_POE_ < FC_Saline_. Absolute values greater than 0.8 are considered “large” effect sizes. Note that edges with large effect sizes are predominantly negative (FC_POE_ < FC_Saline_) with clusters including cortico-cortical and cortico-basal ganglia edges. ***Bottom right panel* D***: **Between-group differences vs. FC_Saline_***. Each point indicates one ROI-to-ROI connection; *x*-values indicate FC_Saline_, and *y*-values indicate FC_POE_-FC_Saline_. The shaded region indicates FC_POE_ < FC_Saline_. The bold line indicates a smoothed curve to visualize overall trends (MATLAB smoothingspline, SmoothingParam = 0.995). Note again that nearly all edges exhibit FC_POE_ < FC_Saline_ and that differences are particularly prominent for “strong” (high FC_Saline_) connections.

In both groups, FC profiles consisted almost entirely of positive (rather than negative) correlations. In both groups, “strong” (high FC) connections occurred in expected well-described resting-state networks (e.g., within sensorimotor cortical networks and between thalamic nuclei; Figures 5A,B). The topology of connectivity (the pattern of which network connections were strong vs. weak) was generally consistent between rats (ANOVA effect of edge: [*p* < 10^−6^; 25.7% of variance explained]), and topology did not grossly vary between groups [no significant group x edge interactions (*p* = 0.56; 2.2% of variance explained)]. In summary, established resting state networks were robustly recapitulated in both study groups.

Between-group comparisons revealed a global reduction in connectivity consistent with multi-network dysfunction and abnormal neural circuitry in POE rats. FC was reduced (FC_POE_ < FC_Saline_) in most network edges examined. Large reductions (Δ < −0.8; Figure 5C) were particularly common in cortico-cortical and thalamo-basal ganglia connections. FC reductions preferentially impacted strong connections (ΔFC = FC_POE_-FC_Saline _≈ −0.1 for connections with FC_Saline _> 0.2 but ΔFC approaching zero for connections with FC_Saline _< 0.2; Figure 5D). Group differences in functional connectivity in specific network edges did not survive multiple comparisons correction. However, in the group x edge ANOVA, brainwide functional connectivity was reduced in the opioid exposed group (effect of group: *p* < 10^−6^; 10.3% of variance explained). In summary, FC was diffusely decreased in POE across cortical and deep gray networks.

## Discussion

While mechanisms of NOWS are well understood, mechanisms of the neurodevelopmental and long-term consequences of POE are still being explored. This is essential given the individual and societal consequences of a growing population of children with lifelong cognitive and behavioral issues stemming from POE. This study supports the growing body of literature that POE has long-term structural and functional neurological sequelae, including lasting brain injury. Specifically, we found that POE resulted in (1) diffuse decreases in large-tract white matter anisotropy and (2) diffuse, widespread decreases in functional connectivity between gray matter regions in adult rats. Previously, using the same model of POE, we identified a robust systemic inflammatory response syndrome and immune system dysfunction during the neonatal period concomitant with microstructural white matter injury and cognitive deficits in adulthood (39). POE led to immune cell priming in the immediate perinatal period with significant baseline elevation in secretion of pro-inflammatory cytokines and chemokines, as well as an exaggerated inflammatory response from PBMCs after stimulation with LPS (40, 41). This effect lasted in adulthood, and included shifts in cerebral immune cell populations, defined specifically by increased neutrophils and regulatory T-cells, occurring months after prenatal opioid exposure (40). The present data extend these findings by confirming structural and functional MRI changes through adulthood, emphasizing the neurodevelopmental care and follow-up that children exposed to opioids need beyond the NICU or formal medical and hospital setting.

### Microstructural alterations

We found decreased fractional anisotropy in large-tract white matter ROIs examined—in keeping with decreases in white matter FA described in human studies of POE to date [in the internal capsule and internal longitudinal fasciculus in term infants (12, 32), and in central inferior and posterior white matter tracts in school-aged children, respectively] (49, 50). These cross-sectional human subjects studies have been unable to attribute these alterations to POE itself as opposed to associated biopsychosocial factors; our results suggests that POE is itself sufficient to decrease white matter FA (37). Underlying architectural differences responsible for differences in diffusion metrics remain unknown; decreased FA may be caused, for instance, by larger axon diameters, by a lower axon packing density, or by increased membrane permeability (whether due to decreased myelination or otherwise) (51). Trends towards decreased axial diffusivity and increased radial diffusivity in POE in this study provide some clues: as diffusivity in b = 1,000 imaging is thought to be driven mainly by extra-axonal water flow, increased radial diffusivity may suggest decreased myelin volume, decreased axonal density, or a loss of extracellular matrix (51). We previously identified white matter volume loss and axonal injury in this model in *ex vivo* pathology that is consistent with these long-term changes in diffusion (39). Taken together with the profound inflammation that is present during this developmental time frame, the effects on the elaborate neurodevelopmental program guiding oligodendrocyte maturation, myelination and neural circuit formation cannot be overemphasized (52, 53).

### Decreased functional connectivity

Our primary fMRI finding was a diffuse decrease in FC in POE. Decreased FC is often interpreted as a decrease in bidirectional information flow between gray matter regions, and such a decrease could be expected in the setting of diffuse white matter alterations. Potential alternative/additional causes of apparent decreases in FC should also be considered—including displacement of functional processing nodes (altered topography) or differences in network constituents (54). The preservation of “neurotypical” topography suggests that atlas-based parcellation remains grossly accurate (e.g., that primary motor cortex is similarly located in both groups), but more sophisticated techniques such as representational similarity analysis would be needed to exclude more subtle topographical or topological differences in network structure (55).

Studies of infants and children with a history of POE have not converged upon a characteristic “signature” of altered functional connectivity in this population (37). As such, it is difficult to compare our findings directly to extant literature. Again, however, this preclinical study may help differentiate effects of POE itself from effects of associated biopsychosocial factors.

### Clinical implications

Neurocognitive sequelae of POE appear to impact a number of cognitive domains—ultimately impacting psychomotor and behavioral outcomes (56–61). Especially in older children, impairments in general cognition, psychomotor development, language development, fine motor skills, hand-eye coordination, attention, and executive function have all been raised as significant concerns (5, 29, 35, 62–69). Children born to opioid-dependent pregnant people have a greater likelihood of being impaired in two or more domains at school entry compared to non-opioid exposed children, and they carry their risk for educational delay throughout their school years (5, 35, 66–69). Impacts on attention and executive function have been particularly prominent. Children with POE are at greater risk for impaired executive function and have difficulties with information processing, and children with POE are at higher risk of developing ADHD (25, 35, 57, 58, 70).

This relatively non-specific pattern of developmental cognitive challenges is common across many neurologic conditions and can result from various brain injury patterns. Deficits in attention and executive function are common in white matter disorders ranging from neurodevelopmental disorders (e.g., spastic cerebral palsy) to acquired brain injury (e.g., traumatic brain injury or multiple sclerosis) (71–73). In each of these disorders, multi-domain cognitive performance (including prominent deficits in attention and executive functioning) has been linked to white matter DTI metrics. The data presented here increases concern that the neurocognitive sequelae of POE may similarly be mediated by diffuse network dysfunction.

This paper adds to a growing body of clinical and preclinical evidence suggesting that neurocognitive sequelae of POE are associated with quantifiable abnormalities in brain structure and in functional connectivity profiles (3, 5, 12, 14, 23, 28, 32, 36). Neonates exposed to methadone or buprenorphine have smaller brains, microcephaly, reduced basal ganglia and cerebellar volumes, reduced cortical thickness, and impaired white matter tract development (23, 32, 49, 50, 74–77). They have microstructural brain injury seen on MRI and impaired neurodevelopment (30, 78, 79). Decreased volumes (whole brain, cortical volume/thickness, and deep gray nucleus) and the white matter DTI profile observed here (decreased FA, decreased AD, and increased RD) have in particular been associated with general cognitive functioning in the POE population (30, 33, 50, 78, 79). However, as highlighted above, further mechanistic evaluation of the effects of methadone and buprenorphine use on the developing brain and long-term outcome studies are desperately needed.

Advances in molecular neuroscience reveal the importance of the multifaceted interplay of the central and peripheral immune systems in regulating brain development and the impacts on dynamic and developing neural circuitry. Indeed, POE occurs at a critical timepoint in development that disrupts the delicate homeostatic pathways essential for proper maturation of neural and neural-immune communication and function (39–41). Recently published data suggest opioid exposure commencing *in utero* propagates inflammation and that POE shares many features of a profound neuroinflammatory disease concomitant with white matter loss and axonal injury (39–41, 80), and immune activation has implications for maladaptive opioid-induced neuroplasticity. Indeed, TLR4 binds microenvironmental toxins, such as LPS and opioids, in both fetal and maternal compartments (81). Methadone can readily cross the placenta and blood-brain barrier and can lead to direct stimulation of inflammatory pathways via TLR4-mediated signaling (82–84). By shifting these pathways towards a pro-inflammatory state, opioids alter the developing immune system, and this alteration is sustained (39, 80, 85). However, how opioids interact with TLR4 in the developing CNS and on immature neural cells is unknown.

From a broader public health perspective, clinical practice guidelines suggest that treating OUD with methadone or buprenorphine is safer than abstinence or withdrawal during pregnancy (65, 86–89). The evidence reviewed above, however, suggests that long-term neurocognitive sequelae are not fully mitigated by replacement strategies and that there is potential for untold consequences on neural cell maturation, circuit formation and plasticity. Beyond mechanistic research, we are hopeful that further preclinical work extending this study may be of use in developing translatable opioid-sparing protocols during pregnancy and in the perinatal period to further prevent neurocognitive sequelae. DTI and FC studies performed in larger cohorts of children, as they mature, would also be beneficial.

### Limitations

This was a single study performed using a single model (one strain of one species with one exposure/dosage). While parallels to changes in brain structure and neurocognitive phenotypes seen in humans following POE are reassuring, it cannot be assumed that brain injury mechanisms are identical to those in human POE or that mechanisms are the same across dosing/dose timing regimens. Opioid exposure in this model occurs from E16 through P21 and may not reflect the effects of opioid exposure early in pregnancy (E0 to E15).

While we included an equal number of males and females in this investigation, our study was not powered to evaluate differences in connectivity based on sex. Further investigations into sex dependent differences, including changes in body size throughout the lifespan with opioid exposure and brain connectivity are important for identifying novel mechanisms of injury at the circuit level, for identifying at-risk individuals, and for evaluating responsiveness to novel therapeutic approaches including neuroimmunomodulation.

*In vivo* imaging protocols used in this study carry potential confounds from artifacts (e.g., motion, cardiac/respiratory pulsation, effects of sedation). We have attempted to mitigate the effects of these artifacts to the degree currently achievable using best practices, but confounding effects remain possible.

## Conclusions

In sum, these studies connect POE to impaired neural maturation, aberrant white matter microstructure, weakened network connectivity, and fragmented neural networks in adulthood. These data emphasize the need for long-term neurodevelopmental follow-up in children with POE. In addition, a critical need exists for novel and precise diagnostic and prognostic imaging and biobehavioral biomarkers, and elucidation of novel druggable targets for neurorepair in this vulnerable patient population. Moving forward, it is essential to understand how *in utero* insults constrain brain structure and function in adulthood, and what targeted interventions will be required to improve long-term outcomes in the countless children born exposed to opioids each year.

## Data Availability

The raw data supporting the conclusions of this article will be made available by the authors, without undue reservation.
